# Correction: Li et al. Multidrug-Resistant *Streptococcus agalactiae* Strains Found in Human and Fish with High Penicillin and Cefotaxime Non-Susceptibilities. *Microorganisms* 2020, *8*, 1055

**DOI:** 10.3390/microorganisms9061198

**Published:** 2021-06-01

**Authors:** Carmen Li, Dulmini Nanayakkara Sapugahawatte, Ying Yang, Kam Tak Wong, Norman Wai Sing Lo, Margaret Ip

**Affiliations:** Department of Microbiology, Prince of Wales Hospital, The Chinese University of Hong Kong, Hong Kong Special Administrative Region (HKSAR), Hong Kong, China; 2carmen.li@cuhk.edu.hk (C.L.); 1155085653@link.cuhk.edu.hk (D.N.S.); yingyang@cuhk.edu.hk (Y.Y.); kamtakwong@cuhk.edu.hk (K.T.W.); normanlo@cuhk.edu.hk (N.W.S.L.)

The authors would like to make the following correction to the published paper [1].

There is a mistake in the last sentence of the second paragraph in Section 3.2 Antimicrobial Susceptibility Testing.

The sentence should read “ARGs conferring resistance to macrolide (mreA) was found in F49”.

This change will not alter the conclusions drawn in the paper.
